# Multifaceted Quality Improvement Initiative Improves Retention in Treatment for Youth with Opioid Use Disorder

**DOI:** 10.1097/pq9.0000000000000174

**Published:** 2019-05-16

**Authors:** Casey B. Cottrill, Stephanie Lemle, Steven C. Matson, Andrea E. Bonny, Erin R. McKnight

**Affiliations:** From the * Nationwide Children’s Hospital, Columbus, Ohio; † The Ohio State University, Columbus, Ohio; ‡ The Research Institute, Nationwide Children’s Hospital, Columbus, Ohio

## Abstract

**Introduction:**

There is a critical need to develop interventions that help adolescents and young adults with opioid use disorders (OUDs) connect with, engage in, and remain consistent with the treatment given that patients who develop long-term OUDs experience long-term medical and mental health sequelae.

**Methods:**

We implemented quality improvement (QI) processes to increase early engagement and 6-month retention within a medication-assisted treatment clinic for youth with OUDs. QI interventions included motivational interviewing (MI) staff training, implementation of reduced initial treatment requirements, reduction of access barriers to treatment, and enhancement of patient treatment motivation. We monitored the impact of the interventions via a p-chart.

**Results:**

A statistically significant shift was seen in the 6-month retention rate following both MI staff training and the use of reduced initial treatment requirements. Second visit return rate also experienced a statistically significant shift following transportation support and an incentive program.

**Discussion:**

Our data demonstrate that following MI staff training, reduced initial clinic requirements, transportation support, and utilization of an incentive program, the second visit return rate, and 6-month retention rate improved within an outpatient medication-assisted treatment clinic for youth with OUDs.

## Abstract 2

Supplemental Digital Content is available in the text.

## INTRODUCTION

Rates of opioid abuse among United States’ adolescents continue to rise at an alarming rate highlighting the need for early recognition and novel treatments of this problem.^1–3^ Among adolescents, deaths from opioids exceed those from all other substances, and for youth who develop a long-term opioid addiction, now termed opioid use disorder (OUD), the outcomes are grim.^4,5^ Given the potentially chronic and life-threatening nature of OUDs, there is a critical need for interventions that help youth connect with, engage in, and remain consistent with substance use disorder treatment (SUDT).

Currently, the American Academy of Pediatrics and the American Society of Addiction Medicine have published practice guidelines and position statements supporting the first-line use of medication-assisted treatment (MAT) with buprenorphine for adolescents and young adults diagnosed with an OUD.^6,7^ At our institution, a Medication-Assisted Treatment of Addiction (MATA) clinic was developed to provide MAT for youth, 16–22 years of age, with OUD. Prior research within the clinic found high rates of MAT compliance and opioid abstinence among youth engaged in our program. However, poor clinic attendance, a proxy for decreased patient commitment to MAT, was the largest barrier to long-term success.^8^

An intensive literature search guided the quality improvement (QI) initiative and focused this project on motivational interviewing (MI) techniques, clinic requirement modification, clinic treatment barriers, and the use of tokens of incentives. MI techniques are effective in treating youth with substance use disorders.^9,10^ Little is known about factors leading to adolescent and young adult discontinuation of SUDT, but in adults, treatment discontinuation was related to concerns about treatment requirements, difficulty trusting staff, and low treatment alliance.^11,12^ Adult SUDT programs that minimize barriers, such as transportation, to access have higher rates of sober behaviors and successful addiction treatment.^11–13^ In our experience, transportation is often a reason for treatment noncompliance, but the impact on treatment retention for adolescent SUDT has not been identified in the literature. Patient motivation also plays an important role in early engagement and compliance. Lack of treatment motivation is an important factor in adult discontinuation, but little is known of the effects of this on adolescent SUDT.^14–16^ Incorporation of tokens of incentive as part of a structured program has been shown to increase patient motivation for participation within adults seeking treatment for substance use disorders.^17–19^ While reviewing the literature, it was notable that many included studies focused on use disorders such as cocaine and alcohol as opposed to primarily focusing on OUDs. The literature regarding MAT for OUDs most commonly occurred in methadone treatment centers, which require a different frequency of patient visits, with daily treatment attendance requirements.

Our project sought to build on current knowledge and demonstrate the application of similar techniques and interventions within a QI initiative in an outpatient MAT program with adolescents and young adults.^20,21^ The aim of this project was to increase patient retention in our MATA clinic for youth with OUDs through staff development, modified initial clinic requirements, transportation provision, and treatment incentive tokens. Initial analysis demonstrated that baseline clinic 6-month retention was 19% for the previous 2 years, and the percentage returning for a second visit was 75%. We endeavored to:

1. Increase the percentage of patients returning for a second clinic visit to 90%.
2. Increase the 6-month MATA clinic retention rate to 40%.

## METHODS

### Setting and Context

The intervention population consisted of adolescents and young adults with OUD who received care in the multidisciplinary MATA clinic. The clinic population is composed of patients 16–22 years of age with approximately 50 active patients at any given time and about 100 new and reestablishing patients evaluated per year. Our clinical team includes a dedicated medical assistant, social workers, and several adolescent medicine physicians. All patients receive a prescription for buprenorphine/naloxone at the first clinic visit and all subsequent clinic visits provided they remain compliant with clinic requirements such as urine drug screens free of all opiates and attendance of substance use disorder counseling. Patients are screened with the point-of-care urine testing for opioids, cocaine, methadone, amphetamines, oxycodone, tetrahydrocannabinol, and buprenorphine. The clinic does not provide substance use disorder counseling on site, but refers patients to facilities in the area to provide this critical portion of treatment. Clinic social workers coordinate with SUDT providers at those facilities to provide consistent treatment messages and updates to all team members.^22^

### Interventions

#### Development of Key Drivers

**Fig. 1.**
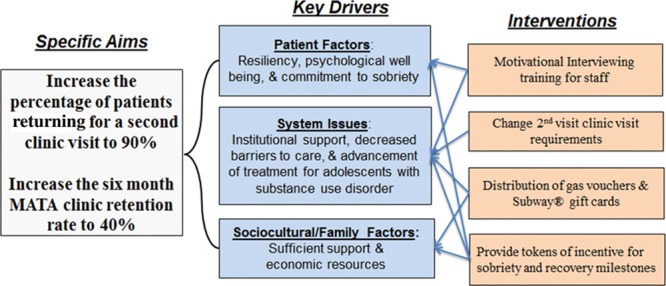
Key driver diagram for QI Initiative. Subway is a registered trademark of Subway IP, LLC © 2018–2019 Subway IP, LLC., Milford, Conn.. All Rights Reserved.

Given our aims of increasing the second visit return rate and 6-month retention rate, our team identified important drivers for change grouped into the patient, system, and family/sociocultural factors. Figure 1 depicts a key driver diagram for the project.

#### Motivational Interviewing

The clinic staff participated in 2 MI training sessions to learn about this specific SUDT tool. These trainings each involved 2 days of intensive group and individual education on the tenets of MI and how to best apply these principles to a population of youth with OUD. Both training opportunities involved instruction by license chemical dependency counselors with specializations in counseling and education. The education involved lecture materials on MI and interactive, case-base practical application of MI in various patient scenarios. To use and maintain the skills learnt, the clinic team discussed MI techniques and stages of change with each patient encounter. These discussions helped the clinic staff to integrate MI strategies into routine management for all patients in our clinic.

#### Initial Clinic Requirements

The clinical team reevaluated the initial requirements given to patients at the first clinic visit to better address system factors. Before this QI initiative, the requirements outlined in the first visit included: a urine drug screen free of opiates, completion of a full drug and alcohol assessment at a SUDT facility, and attendance of at least one local 12-step meeting. MAT was initiated at the first visit and then continued at subsequent encounters as long as the patient remained compliant with clinic requirements. Recognizing that these initial clinic requirements are overwhelming and acknowledging that many patients present in active opioid withdrawal to their initial visit, the team formulated a change to this process. To create a less stressful experience, the team lessened initial treatment requirements for all patients to return to the second visit and strongly encouraging, rather than requiring, a urine drug screen free of opiates. This change provided an early goal attainment opportunity to begin to foster the patient–clinic relationship and facilitated subsequent treatment planning conversations occurring with patients no longer experiencing active opioid withdrawal. Additionally, this transition to reduced initial treatment requirements aligned with the MI tenets of empathetic interactions, meeting the patient where they are at, and rapport building.^23^

#### Transportation and Food Resources

To address sociocultural factors that impact access to care and economic resources, we gave patients food and transportation support. A $10 gas voucher was distributed to all patients to facilitate transportation to their return clinic visit. Gas vouchers provided were for a national chain of gas stations that had numerous locations near the clinic. Patients could either use the voucher themselves or could use this to purchase gas if another person transported them to the clinic. The clinic population is both urban and rural with the average clinic patient traveling an hour to attend an appointment. The gas voucher amount, depending on gas prices, would either completely or mostly cover the cost of gas to attend an appointment. Additionally, all patients received a $10 Subway gift card to help address potential food insecurities at all visits due to limited economic resources. Recognizing that access barriers and resource limitations are not confined to the first return visit, patients continued to receive a $10 gas voucher and Subway gift card at each clinic visit to improve and maintain treatment engagement. We distributed the gas vouchers and Subway gift cards from April to September 2015.

#### Tokens of Incentive

The last intervention addressed all key drivers identified through the use of tokens of incentive for treatment and recovery milestones. Tokens of incentive were rewards attainable through a grab-bag approach that we outlined in the Supplemental Digital Content 1, http://links.lww.com/PQ9/A86.^17–19,24^ We distributed the grab-bag incentives from June to September 2015. During this QI initiative, the per-patient grab-bag earnings ranged from as high as $260 (one $250 gift card and one $10 gift card) to only a book token. The average monetary earnings per visit were approximately $34 per patient, and most visits included the selection of at least one book token.

### Study of the Intervention

The study period was from Q1 2014 to Q4 2016. We collected preintervention baseline data from Q1 2012 to Q4 2013 at the start of the QI initiative. All patients receiving buprenorphine/naloxone at this institution are managed through the MATA clinic so that all of these clinic’s patients could participate.

### Measures

Two outcome measures were chosen: second visit return rate and 6-month retention rate. The second visit return rate measured the percentage of patients who come to an initial MATA clinic appointment and then returned for a second clinic visit 1–2 weeks later. This measure reflects the early engagement in treatment. To better measure, the progression from early engagement to early remission, the 6-month retention rate was utilized as the other outcome measure. OUD in early remission begins after 3 months of sobriety and no longer meeting any of the OUD diagnostic criteria, except continued cravings.^20,21,25^ Six-month retention served as a proxy for continued early remission, as a patient would have met early remission diagnostic criteria for 3 months.^20,21,25^ We measured the 6-month retention rate by the following formula:


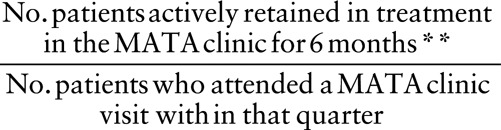


### Analysis

Data were collected quarterly Q1 2014–Q4 2016 from the electronic medical record and results were documented on a proportion chart (p-chart).^26^ Patients were counted in the quarter in which they started attending appointments in the MATA clinic, but not classified as actively retained until 6 months had passed; therefore, data reporting lags 6 months behind real time to allow for appropriate classification of actively retained (ie, patient started in January 2015; retention cannot be measured until July 2015). We reviewed charts manually for any patient with a gap of 6–8 weeks in between appointments to determine the status of actively retained or not actively retained. Of note, having one urine drug screen positive for opioids, amphetamines, benzodiazepines, and tetrahydrocannabinol did not automatically remove a patient from the actively retained category. However, a patient presenting to 3 consecutive appointments with drug screens positive for opioids and/or negative for buprenorphine did lead to treatment discontinuation as per clinic protocol. As addiction is a chronic relapsing disease, some patients left treatment and reengaged during the time of measurement. Each new start to the MATA clinic reset the time to 6-month retention for data analyses to avoid duplicate counting in the data.

### Funding and Ethical Considerations

National Institutes of Drug Abuse training grant in conjunction with the Society for Adolescent Health and Medicine funded this QI initiative. The Institutional Review Board at the participating institution deemed this project QI and not human subjects research. Therefore, it did not require review and approval.

## RESULTS

**Fig. 2.**
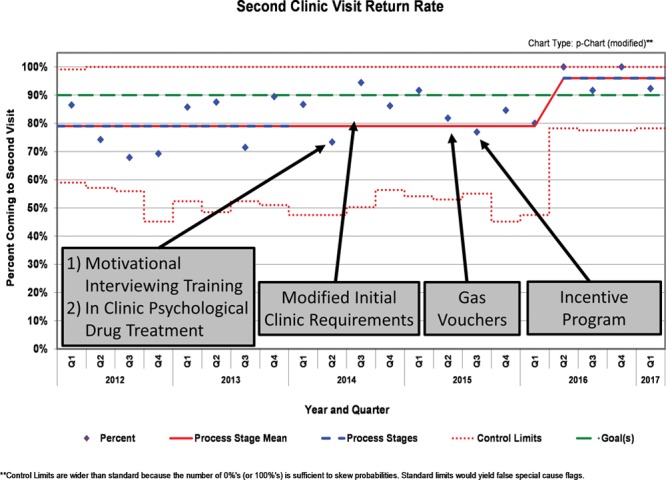
Annotated second visit return rate control chart. **Control limits are wider than standard because the number of 0%’s (or 100%’s) is sufficient to skew probabilities. Standard limits would yield false special cause flags.

A p-chart documented results for the second visit return rate and 6-month retention rate. An annotated p-chart has been provided noting all of the interventions utilized (Fig. 2). The interventions began in Q2 2014 with the MATA clinic providers undergoing MI training. The implementation of reduced initial treatment requirements occurred in Q3 of 2014. We began distribution of gas vouchers, and treatment incentive tokens began in Q2 2015 and completed distribution in Q3 2015 with the consumption of all grant dollars. We are still currently tracking results, but due to the time lag in reporting 6-month retention data, we report 6-month retention rate data through Q4 of 2016 and the second visit return rate through Q2 of 2017.

**Fig. 3.**
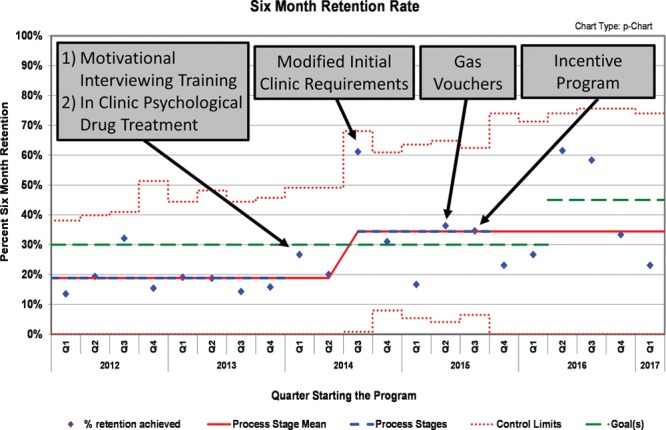
Six-month retention rate annotated control chart.

Six-month retention rate experienced a shift in Q3 2014 (*P* < 0.001) when the mean rate rose from 19% to 34%. Interventions occurring before this shift included the MI training and the implementation of reduced initial treatment requirements. Figure 3 shows these data.

The second visit return rate also experienced a shift in Q2 2016 (*P* < 0.001) when the mean rate increased from 79% to 97%, following the implementation of grant funding to support transportation assistance and incentive tokens. Figure 2 reports these data.

There may have been some external influences on retention and return rates apart from the QI interventions in this initiative. Within the MATA clinic, there was a period when we offered in-clinic substance use disorder counseling in conjunction with local drug treatment facility. This counseling allowed for both MAT and substance use disorder counseling to reside in the same location, thereby decreasing barriers to treatment participation. In the clinic, counseling began in Q2 2014 and lasted through Q3 2016 reflected in Figure 2. Mitigating the impact of this contextual element is the fact that only 4 patients participated in substance use disorder counseling through this specific program.

## DISCUSSION

In this study, we observed statistically significant increases in the 6-month retention rate and the second visit return rate. For the 6-month retention rate, this increase occurred following the MI training and modification of initial treatment visit requirements. Although the individual effects of these 2 interventions cannot be determined, the combination led to an increase in an important recovery milestone, early remission. In a population still undergoing neurocognitive and structural brain development, adolescent and young adult patients are especially vulnerable to continuing substance misuse; therefore, it is imperative to identify interventions that stimulate and achieve early remission.^15,27–31^

The data for the 6-month retention rate do demonstrate variability starting Q4 2015. This variability may be due to both internal and external factors and support the need for long-term investigations of interventions. The lessening of initial treatment requirements may have captured patients who were earlier in the treatment process and may have more difficulty remaining consistent with treatment recommendations leading to retention variability. Additionally, the transportation support and token incentive program ended in September 2015. Being able to provide this support consistently would give a better indication of consistency and long-term impact of the intervention on retention in a MAT program.

The second visit return rate improvement followed the distribution of transportation support and treatment incentive tokens. Although the literature speaks to the need to minimize transportation barriers, and the utilization of a grab-bag incentive technique, to improve adult treatment adherence,^11^ this QI initiative demonstrates a similar need in adolescent and young adult SUDT. The significant increase in the second visit return rate adds to the literature by highlighting the importance of removing treatment barriers, such as transportation difficulties, and incentivizing treatment and recovery milestones within the treatment of OUDs in youth.

The major limitation of this initiative is the small sample size with each quarter analyzed including only 12–26 patients. These low sample sizes lead to less generalizability, but this information remains useful for those operating a MAT clinic for youth due to the paucity of literature focused on QI in this population. Additionally, it would strengthen the study to monitor for changes in 1-year retention rates to better reflect the long-term impact of intervention effects and sustained remission. The MI training and implementation would have benefited from further standardization and monitoring to verify all patients received consistent and authentic MI techniques during each visit. We cannot fully assess the relationship between MI and engagement or retention in a MAT program for youth until verification of MI usage occurs. Therefore, we are limited to discussing the effects of MI training on the outcome measures. Last, a training grant funded the gas vouchers and incentive tokens and funds expired after only 2 quarters during 2015 (Q2 and Q3). Ideally, this intervention would have been implemented over a longer period to more fully assess for special cause variation in retention rates, especially in light of the small sample sizes.

To more accurately evaluate the true impact of these QI interventions, future studies should investigate variations of these interventions to define the relationship between specific interventions and outcome measures better. Specifically, investigations of the grab-bag technique with variations of gift card amounts, goal setting techniques, and incentive distribution will expand the literature and give providers a better understanding of how to best utilize incentives within the treatment of youth with substance use disorders. Additionally, structuring a researched-based study to include randomization and control group methodology will potentially allow for causal relationships to be identified for individual interventions. However, this initiative has demonstrated the feasibility of and potential positive impact of utilizing QI methodology and intervention to address the growing problem of OUDs in youth. This study provides the first steps in a conversation to guide adolescent and young adult treatment providers toward effective QI intervention methods that may support patients through initial SUDT.

## ACKNOWLEDGMENTS

The funding organizations for the training grant had no role in the design and conduct of the study; collection, management, analysis and interpretation of the data, preparation, review, or approval of the manuscript; and decision to submit the manuscript for publication.

## DISCLOSURE

The authors have no financial interest to declare in relation to the content of this article.

## Supplementary Material
